# Inhibition of TLR4 mitigates sensorineural hearing loss resulting from cochlear inflammation

**DOI:** 10.1186/s10020-025-01219-0

**Published:** 2025-05-05

**Authors:** Jintao Lou, Fan Wu, Wei Liu, Rui Hu, Wuhui He, Yisi Feng, Yan Huang, Jia Guo, Jingman Deng, Zhen Zhao, Zhigang Zhang, Yu Si

**Affiliations:** 1 Department of Otolaryngology, Sun Yat-Sen Memorial Hospital, Sun Yat-Sen University, No.107 Yanjiang West Road, Yuexiu District, Guangzhou, 510120 China; 2 Institute of Hearing and Speech-Language Science, Sun Yat-Sen University, Guangzhou, China; 3 Guangdong Provincial Key Laboratory of Malignant Tumor Epigenetics and Gene Regulation, Sun Yat-Sen Memorial Hospital, Sun Yat-Sen University, Guangzhou, Guangdong China; 4 Second Affiliated Hospital, Zhejiang University School of Medicine, Hangzhou, China; 5 Guangdong Provincial Key Laboratory of Cancer Pathogenesis and Precision Diagnosis and Treatment Shenshan Medical Center, Sun Yat-Sen Memorial Hospital, Sun Yat-Sen University, Shanwei, China

**Keywords:** Cochlea, Sensorineural hearing loss, TLR4, Inflammation

## Abstract

**Background:**

Inflammation is a principal cause of sensorineural hearing loss resulting from cochlear injury. However, current research investigating the mechanisms of sensorineural inflammatory injury remains inadequate.

**Methods:**

Cochlear inflammation was induced by administering lipopolysaccharide (LPS) into the otic bulla (OB) and posterior semicircular canal (PSCC). Auditory brainstem responses (ABR) were recorded, and cochlear tissue alterations were analyzed using hematoxylin and eosin (HE) staining and immunofluorescence. Levels of cochlear inflammation were quantified using a cytokine array. Additionally, Toll-like receptor 4 (TLR4) knockout mice were employed to evaluate sensorineural neuroprotection.

**Results:**

LPS injection into the PSCC caused more pronounced and stable cochlear inflammatory damage compared to injection into the OB. LPS exposure led to significant loss of cochlear hair cells, atrophy of the stria vascularis, and spiral ganglion damage. Furthermore, LPS treatment upregulated TLR4 receptor expression, increased the number of Ionized calcium-binding adapter molecule 1 (IBA1) positive cells, and elevated levels of inflammatory cytokines in the cochlea. TLR4 knockout (TLR4-KO) mice demonstrated reduced LPS-induced cochlear sensorineural damage.

**Conclusion:**

LPS injection into the PSCC induces sensorineural tissue damage in the cochlea and results in sensorineural hearing loss. These findings suggest that TLR4 inhibition can alleviate cochlear inflammation-induced sensorineural hearing loss. TLR4 represents a potential therapeutic target for sensorineural hearing loss.

## Background

Approximately 20% of the global population experiences some degree of hearing loss, with a substantial portion attributed to irreversible sensorineural hearing loss (Wilson and Tucci 2021). The etiology of sensorineural hearing loss is multifactorial, involving genetic mutations, noise exposure, ototoxic drugs, infections, and aging (Cunningham and Tucci 2017; Lieu et al. 2020). Infections in the middle or inner ear can provoke inflammatory responses that damage sensory nerve tissue in the cochlea, ultimately resulting in hearing loss (Lundman et al. 1992; Guo et al. 1994; Engel et al. 1998). Given the lack of effective clinical treatments for sensorineural hearing loss, advancing research into its pathogenesis is of critical importance.

Our preliminary studies revealed that patients clinically diagnosed with otitis media caused by Gram-negative bacterial infections are at a higher risk of developing sensorineural hearing loss (Lou et al. 2024). LPS, a major virulence factor of Gram-negative bacteria, is commonly used to induce inflammatory damage in sensory nervous systems, including those of the eyes, nose, and brain. Therefore, we sought to further investigate the mechanisms underlying LPS-induced hearing impairment (Ren et al. 2019; Tiboc Schnell et al. 2021; Peng et al. 2023). To this end, we utilized LPS injections into the middle and inner ear to replicate infectious inflammatory injury and explore the mechanisms of cochlear damage.

Sensorineural hearing loss caused by cochlear injury primarily affects the hair cells, stria vascularis, and spiral ganglia neurons (SGNs). Hair cells, as sensory epithelial cells, detect sound vibrations and transduce them into neural signals, thereby playing a critical role in auditory function (Fettiplace and Hackney 2006; Hudspeth 2014). The stria vascularis, located in the cochlear lateral wall and connected to the spiral ligament, contains abundant capillaries essential for producing endolymph—a fluid crucial for maintaining normal cochlear function and auditory signal conduction (Zhang et al. 2021). The stria vascularis supports the production and secretion of endolymph, which helps preserve the cochlea's internal environment. Damage or dysfunction of the stria vascularis can impair endolymph production, ultimately leading to hearing loss. Hair cells convert the mechanical energy of sound into nerve impulses, which are transmitted to the auditory center through connections with SGNs. SGNs are classified into two types: type I cells, which synapse with inner hair cells to transmit sound signals to the brain, and type II cells, which synapse with outer hair cells and are involved in conveying directional and intensity-related information (Liberman and Kujawa 2017; Kobel et al. 2017). The proper functioning of spiral nerves is essential for sensorineural hearing, and damage to these nerves caused by cochlear injury can result in sensorineural hearing impairment. This study, therefore, focuses on examining changes in hair cells, stria vascularis, and SGNs functions using an animal model of cochlear injury.

Toll-like receptor-mediated inflammatory processes play a crucial role in innate immune responses. Among these, the TLR4 receptor is a central component of the LPS signaling pathway, recognizing and binding LPS to activate downstream signaling molecules. Extensive research has demonstrated that LPS upregulates TLR4 expression, thereby enhancing the secretion of pro-inflammatory factors and exacerbating inflammation (Alan et al. 2022; Radzyukevich et al. 2023). Additionally, multiple studies have consistently shown that inhibiting TLR4 effectively alleviates LPS-induced tissue damage (Li and Feng 2022; Wang et al. 2021; Qi et al. 2023). Based on these findings, we hypothesize that TLR4 serves as a critical regulator of LPS-induced inner ear damage. To investigate this, we utilized TLR4-KO mice to evaluate its role in mediating LPS-induced sensorineural damage in the cochlea.

In our study, we established a stable model of infection-induced inflammatory injury in the cochlea, characterized the phenotype of sensory nerve tissue damage, and investigated the critical role of TLR4 in mediating inflammatory injury.

## Methods

### Animals

All wild-type (WT) mice used in this study were healthy 6- to 8-week-old male C57BL/6 mice obtained from the Experimental Animal Center of Sun Yat-sen University. TLR4-KO homozygous mice were purchased from Shanghai Southern Model Organisms Science & Technology Development Co., Ltd. All experimental animals were housed in quiet, clean, and controlled environments with temperatures maintained at 20–26 °C and a 12-h light/dark cycle. This experiment utilized a total of 45 mice, all of which were performed within these facilities. All mice were anesthetized with a mixture of ketamine (100 mg/kg) and xylazine (10 mg/kg).

### Identification of mouse genotyping

DNA was extracted from genetically modified mice using a mouse gene extraction kit (D0065S, Beyotime, China). The extracted DNA was mixed with corresponding primers and amplified according to the manufacturer's instructions for the Green Taq Mix kit (P131, Vazyme, China). The primer sets were designed as follows: mutant primer AGCAAAGACAAGGGAGTAAGAA (5'→ 3'), WT primer GTCCCTGATGACATTCCTTCT (5'→ 3'), and common primer CTGTTTCTTGCCCATAGTTGA (5'→ 3'). The amplification products were approximately 600 base pairs for the mutant fragment and 793 base pairs for the WT fragment. Amplified products were then analyzed using gel electrophoresis.

### Cochlear inflammatory injury model

The mice were anesthetized and positioned laterally under a microscope on the operating table. After placing a surgical drape, the area behind the left ear was exposed, and hair was removed using hair removal cream. The surgical site was then disinfected. A posterior ear incision, approximately 0.5–1 cm behind the mouse’s ear, was made parallel to the ear. The subcutaneous tissue and muscle were carefully separated to expose the PSCC or OB. A hole was drilled at the center of the PSCC or OB, and a 0.1 mm PE tube was inserted into the drill hole. Subsequently, 10 μL of normal saline (NS) or 1 mg/mL LPS solution (L2880, Sigma-Aldrich, USA) was injected into the OB (Chai et al. 2021). For the PSCC, 1 μL of saline or 1 mg/mL LPS solution was injected at a rate of 5 nL/s using a microinjection pump (UMP3, WPI, USA). After the injection, the drill hole was sealed with muscle, and the skin was aligned and sutured following the application of bio-gel. The incision site was disinfected, and the mice were placed on a constant temperature heating pad until they regained consciousness.

### Auditory testing

ABR measurements were performed using the Tucker-Davis Technologies (TDT System III, Alachua, FL, USA) before treatment and at 3, 7, and14 days after treatments. Subdermal needle electrodes were placed at the vertex (active), under the left ear (reference), and under the right ear (ground). Auditory stimuli were presented as 10-ms (ms) tone bursts with a 1 ms rise/fall time at frequencies of 8, 16, and 32 kHz. The average response to 1000 stimuli was recorded while the sound intensity was progressively reduced in 10 dB intervals from 100 dB SPL. The ABR threshold was defined as the lowest stimulus intensity that elicited a reproducible waveform response.

### Histological preparations

The mouse cochlea was dissected and fixed overnight in 4% paraformaldehyde (BL539 A, Biosharp, China) at 4 °C. After three washes in PBS, the samples were decalcified in EDTA decalcifying solution (E1171, Solarbio, China) at 4 °C for 24 h. Under a stereomicroscope, the basilar membrane was isolated from the cochlea and divided into apical (70–100% from the base), middle (30–70% from the base), and basal (0–30% from the base) regions. For cochlear section preparation, the cochlea was dehydrated and embedded in paraffin. Sections 4 µm thick were collected and subjected to either Hematoxylin–Eosin (HE) staining or immunofluorescence staining.

### HE staining

The paraffin sections were placed in an oven at 60 °C for 1 h. Subsequently, the sections were dewaxed by sequential immersion in xylene and graded alcohol. After HE staining, the sections were dehydrated again through graded alcohol and xylene. Finally, the sections were dried, mounted, and imaged under a microscope.

### Immunofluorescence

Basilar membranes and cochlear sections were permeabilized with 1% Triton X-100 (T8200, Solarbio Life Sciences, China) for 30 min and blocked with 10% bovine serum albumin (BSA) (4240GR100, Biofroxx, Germany) for 30 min at room temperature. The samples were then incubated with primary antibodies overnight at 4 °C. The following primary antibodies were used: rabbit polyclonal anti-myosin 7a (1:500; 25–6790, Proteus Biosciences, China), rabbit purified anti-Tubulin β3 (TUJ1) (1:100; 801,202, BioLegend, USA), and rabbit anti-Iba1 (1:200; ab178846, Abcam, USA). After incubation, the samples were washed three times with PBS and subsequently incubated with secondary antibodies (1:200; 8889, 4412, 8890, Cell Signaling Technology, USA) for 2 h at room temperature. Finally, the samples were washed three times with PBS, mounted with DAPI (ab285390, Abcam, USA), and imaged using a semi-automatic inverted fluorescence microscope (Olympus IX73, Japan).

### Cytokine array kit analysis

We collected the cochleae on the surgical side and placed them in sterile NS on ice. After removing extracochlear tissues and washing three times, we removed the bony capsule, collected the intracochlear tissues, rapidly froze them in liquid nitrogen, and stored them. Cochlear tissue proteins were extracted (7 cochleae/group). A mouse cytokine array kit (R&D Systems, Cat #ARY006) was used to simultaneously detect 40 mouse cytokines in LPS-treated cochlear samples according to the manufacturer’s instructions. Chemiluminescent signals from each membrane were captured using an Amersham Imager 600 (GE Healthcare Life Sciences, Pittsburgh, PA). The intensity (pixel density) of each spot on the membrane was quantified using ImageJ software, corrected for background intensity, and normalized to the positive control on the same membrane.

### Statistical analyses

Statistical data analysis and chart generation were performed using SPSS v26.0 and GraphPad Prism v9.0, while fluorescence images were processed, and fluorescence intensity was calculated using ImageJ software. Statistical analysis was performed using data from three biological replicates, with technical replicates averaged for each biological replicate before analysis. ABR thresholds, hair cell counts, stria vascularis thickness, and IBA1^+^ cells were conducted using two-way analysis of variance (ANOVA), and Tukey's Honestly Significant Difference (HSD) test was used for post—hoc analysis of inter—group differences. TLR4 positive expression and SGNs were statistically analyzed by non-parametric test. A *P*-value of ≤ 0.05 was considered statistically significant.

## Results

### Changes in hearing thresholds were induced by LPS injection in both OB and PSCC

**Fig. 1:**
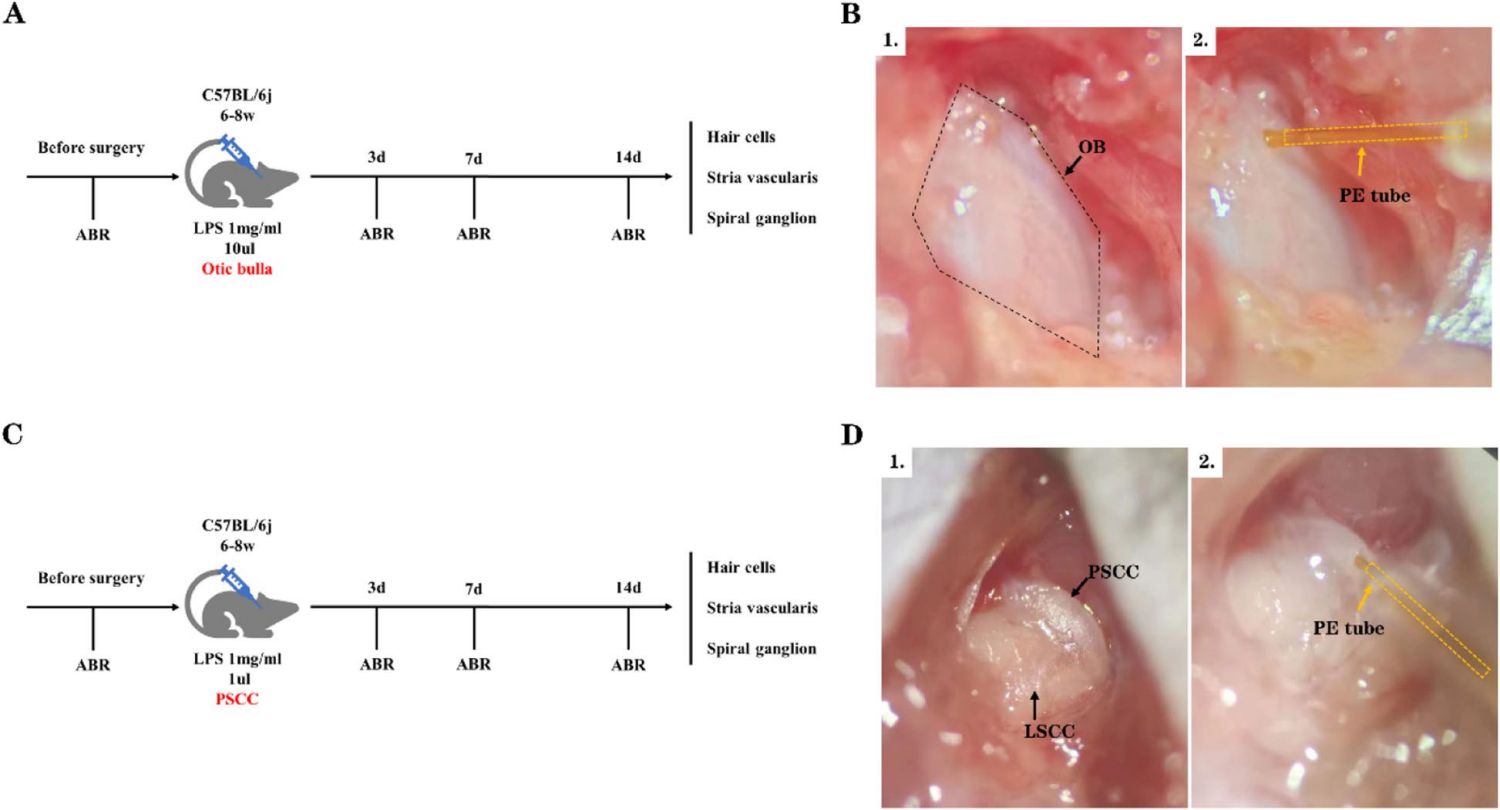
LPS Injection into the Mouse Cochlea. **A** Timeline illustrating the monitoring process for LPS injection into the OB. **B** Schematic representation of LPS injection into the OB, with the black dashed line outlining the OB and the yellow dashed line indicating the polyethylene (PE) tube. **C** Timeline illustrating the monitoring process for LPS injection into the PSCC. **D** Schematic representation of LPS injection into the PSCC; arrows point to the lateral semicircular canal (LSCC) and PSCC

The aim of this study was to determine whether auditory alterations occurred in mice following the injection of LPS into the OB and PSCC. ABR thresholds were measured prior to surgery and on days 3, 7, and 14 post-surgeries. The tympanic cavity was accessed by drilling into the OB, and 10 μL of LPS at a concentration of 1 mg/mL was injected into the cavity. The method for PSCC drilling and injection followed the approach described by Zhao et al (Zhao et al. 2022) (Fig. 1).

**Fig. 2:**
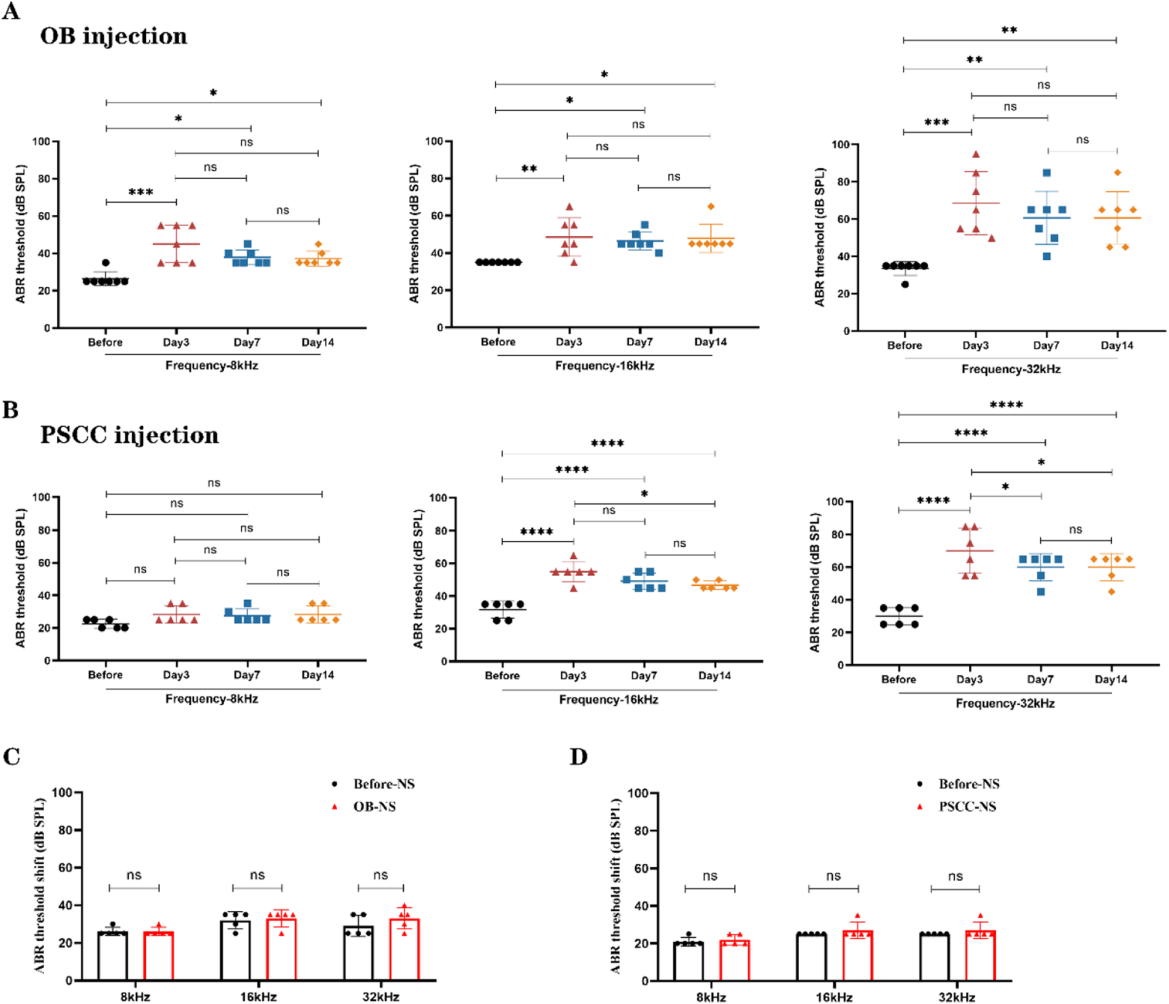
Hearing threshold changes in mice Induced by LPS Injection into the OB and PSCC. **A** LPS injection into the OB resulted in significant threshold shifts at various time points for ABR thresholds at 8, 16, and 32 kHz (*n* = 7, biological replicates). **B** LPS injection into the PSCC did not result in significant threshold changes at 8 kHz compared to the preoperative level. However, more pronounced threshold shifts were observed at higher frequencies of 16 and 32 kHz (*n* = 6, biological replicates). **C** No significant changes in hearing thresholds were observed 7 days after NS injection into the OB (*n* = 5, biological replicates). **D** No significant changes in hearing thresholds were observed 7 days after NS injection into the PSCC (*n* = 5, biological replicates). All data are presented as mean ± SD. Statistical significance: *p < 0.05, **p < 0.01, ***p < 0.001, ****p < 0.0001; ns: no statistical difference

In the OB injection group, significant ABR threshold shifts were observed at 8 kHz, 16 kHz, and 32 kHz on postoperative days 3, 7, and 14 compared to preoperative hearing levels. No significant differences (ns) were detected between postoperative day 14 and day 7 (Fig. 2A). The most pronounced threshold shift occurred on postoperative day 3, with no further changes observed between day3, day 7 and day 14.

In the PSCC injection group, no significant changes (ns) in ABR thresholds were observed at 8 kHz on postoperative days 3, 7, and 14 compared to preoperative levels. However, significant threshold shifts were detected at 16 kHz and 32 kHz. Across all frequencies, no significant differences were observed between postoperative day 7 and day 14 (Fig. 2B). Based on these findings, all subsequent experiments were performed 7 days after LPS injection.

To assess whether the surgical procedure itself caused cochlear damage and affected hearing, we injected NS into the OB or PSCC. On postoperative day 7, no significant differences (ns) were observed in the average ABR thresholds at 8 kHz, 16 kHz, and 32 kHz before and after NS injection (Fig. 2C-D).

### Significant hair cell loss in the cochlear inflammatory injury model

**Fig. 3:**
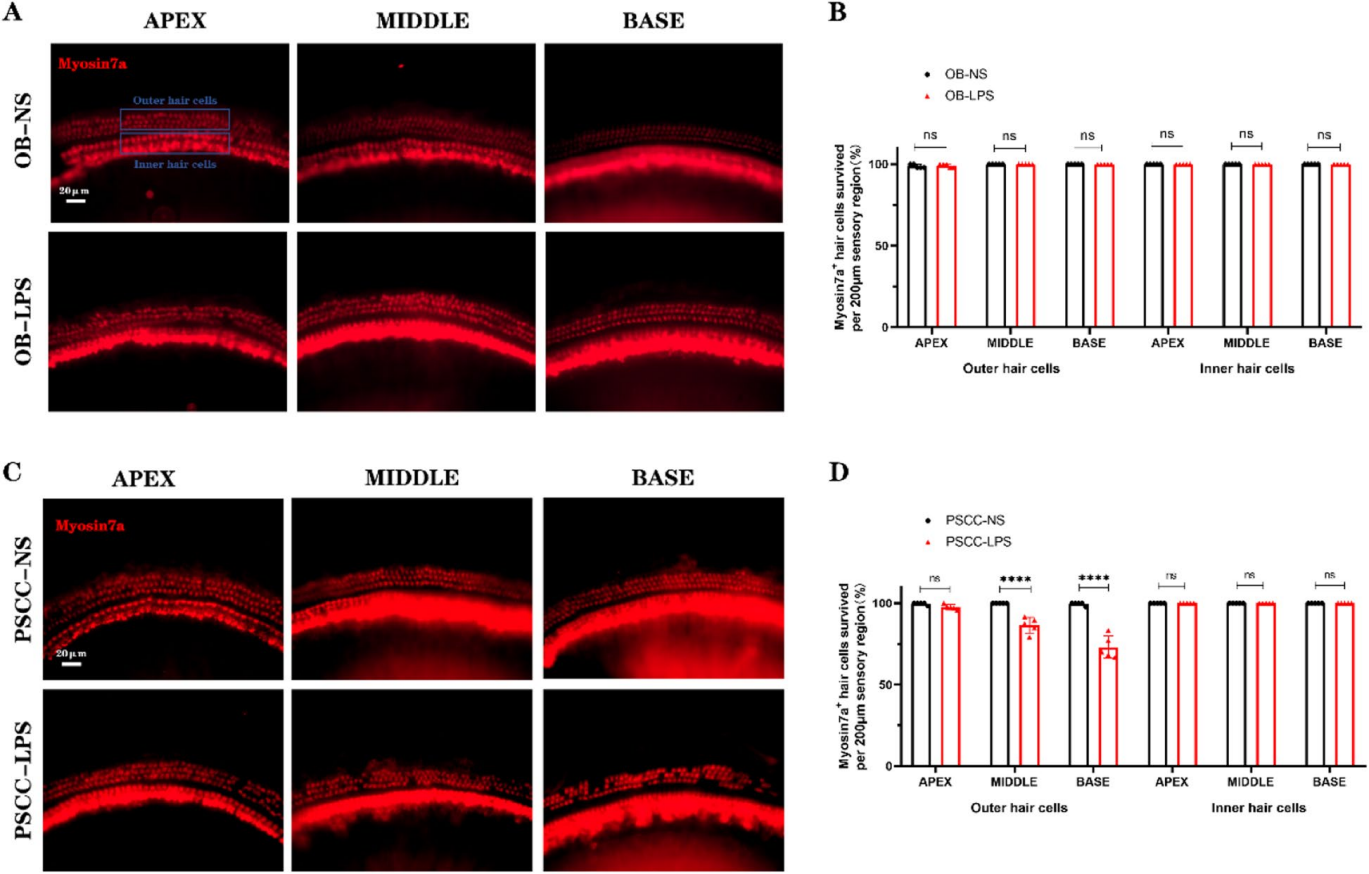
Significant hair cell loss following LPS Injection into the PSCC. **A**. Myosin7a staining of mouse cochlear hair cells after LPS injection into the OB. The blue box indicates the location of the outer and inner hair cells. **B** No significant hair cell loss was observed after LPS injection into the OB (*n* = 5, biological replicates). **C** Myosin7a staining of mouse cochlear hair cells after LPS injection into the PSCC. **D** Significant outer hair cell loss was observed in the middle and basal turns of the basilar membrane following LPS injection into the PSCC (*n* = 5, biological replicates). Scale bar: 20 μm. All data are presented as mean ± SD. ****p < 0.0001; ns: no statistical difference

The ABR hearing results remained stable after LPS injection. Subsequently, anatomical patches were applied to the cochlear basement membrane, and hair cell loss was assessed using immunofluorescence staining with Myosin7a, a hair-cell-specific antibody (Fig. 3A). Compared with the injection of NS into the OB, there was no significant loss of hair cells in the basilar membrane of the cochlea injected with LPS. Additionally, no differences were observed in hair cell counts between the two groups (ns) (Fig. 3B). Following LPS injection into the PSCC, anatomical patches were similarly applied to the cochlear basement membrane, and immunofluorescence staining was performed to examine cochlear hair cell loss (Fig. 3C). In the NS PSCC injection group, no notable hair cell loss was detected, and hair cells appeared neatly arranged and intact. In the LPS PSCC injection group, the survival rate of apical hair cells was 97.917 ± 2.085%, with no statistically significant difference from the NS PSCC injection group (ns). However, in the middle turn, the survival rate of hair cells was 86.113 ± 6.364%, which was significantly lower than in the NS PSCC injection group (** *p* < 0.01). In the basal turn, the survival rate of hair cells was 75.693 ± 8.416%, showing a highly significant difference compared to the NS PSCC injection group (****: *p* < 0.0001) (Fig. 3D).

### Evident vascular atrophy in cochlear inflammatory injury model

**Fig. 4:**
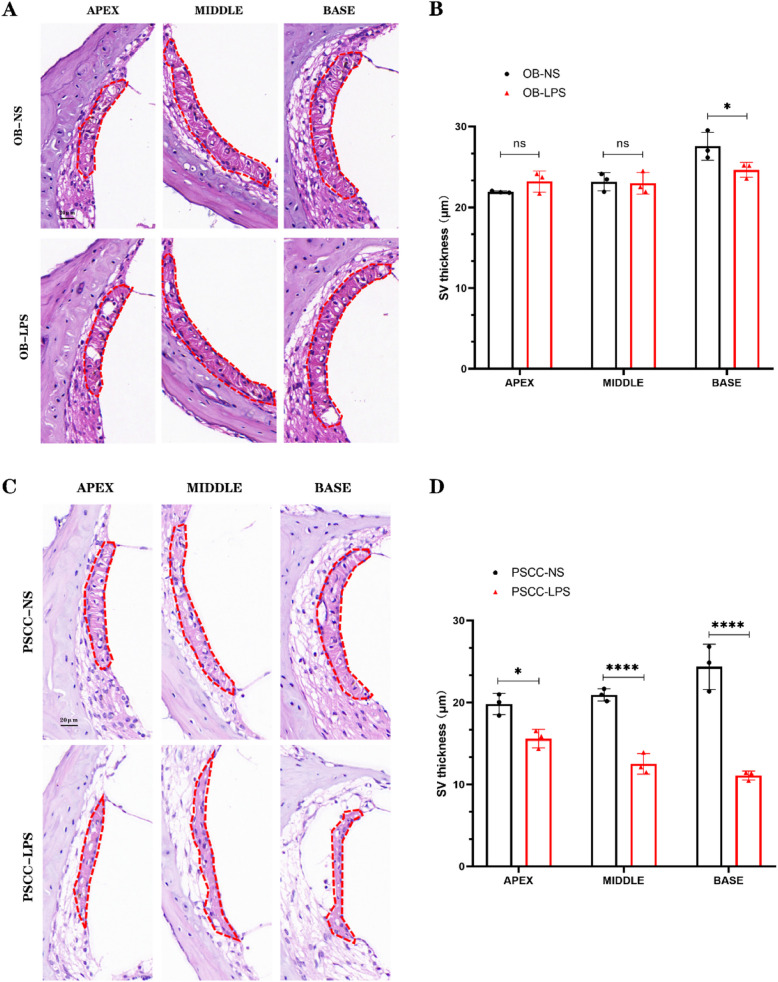
Significant atrophy of stria vascularis after injection of LPS into the PSCC. **A** Vascular pattern of the cochlea in mice after LPS injection, with the red dashed line indicating the stria vascularis of the cochlear lateral wall. **B** Only significant changes in the thickness of the basal stria vascularis were observed after injection of LPS into the OB. **C** The morphology of the stria vascularis in the cochlear lateral wall of mice exhibited significant alterations following LPS injection into the PSCC. **D** The stria vascularis showed significant atrophy after LPS injection into the PSCC. Scale bar: 20 μm. (*n* = 3, biological replicates, each with 3 technical replicates, all data are presented as mean ± SD. *p < 0.05, ****p < 0.0001; ns: no statistical difference)

The stria vascularis of the cochlear lateral wall is a critical tissue responsible for maintaining the equilibrium between the inner ear's electrical potential and the potassium ion concentration in the endolymph and perilymph of cochlea. To investigate morphological alterations in the stria vascularis, paraffin sections of the cochlea were prepared after OB injection, followed by HE staining. The results showed that there was no significant difference in the thickness of the stria vascularis between the apical and middle turns of the cochlea between the NS OB injection group and the LPS OB injection group (ns). However, the stria vascularis of the basal turn in the LPS OB injection group exhibited slight atrophy compared to the NS OB injection group (* *p* < 0.05) (Fig. 4A-B). Significant morphological changes in the stria vascularis on the cochlear lateral wall were observed after PSCC injection (Fig. 4C). In the LPS PSCC injection group, the stria vascularis at the apex, middle, and basal turns showed notable atrophy. The difference in stria vascularis thickness at the apical turn between the two groups was significant (* *p* < 0.05), while the differences in the middle and basal turns were highly significant (**** *p* < 0.0001) (Fig. 4D).

### Damage to the cochlear SGNs induced by cochlear inflammatory injury

**Fig. 5:**
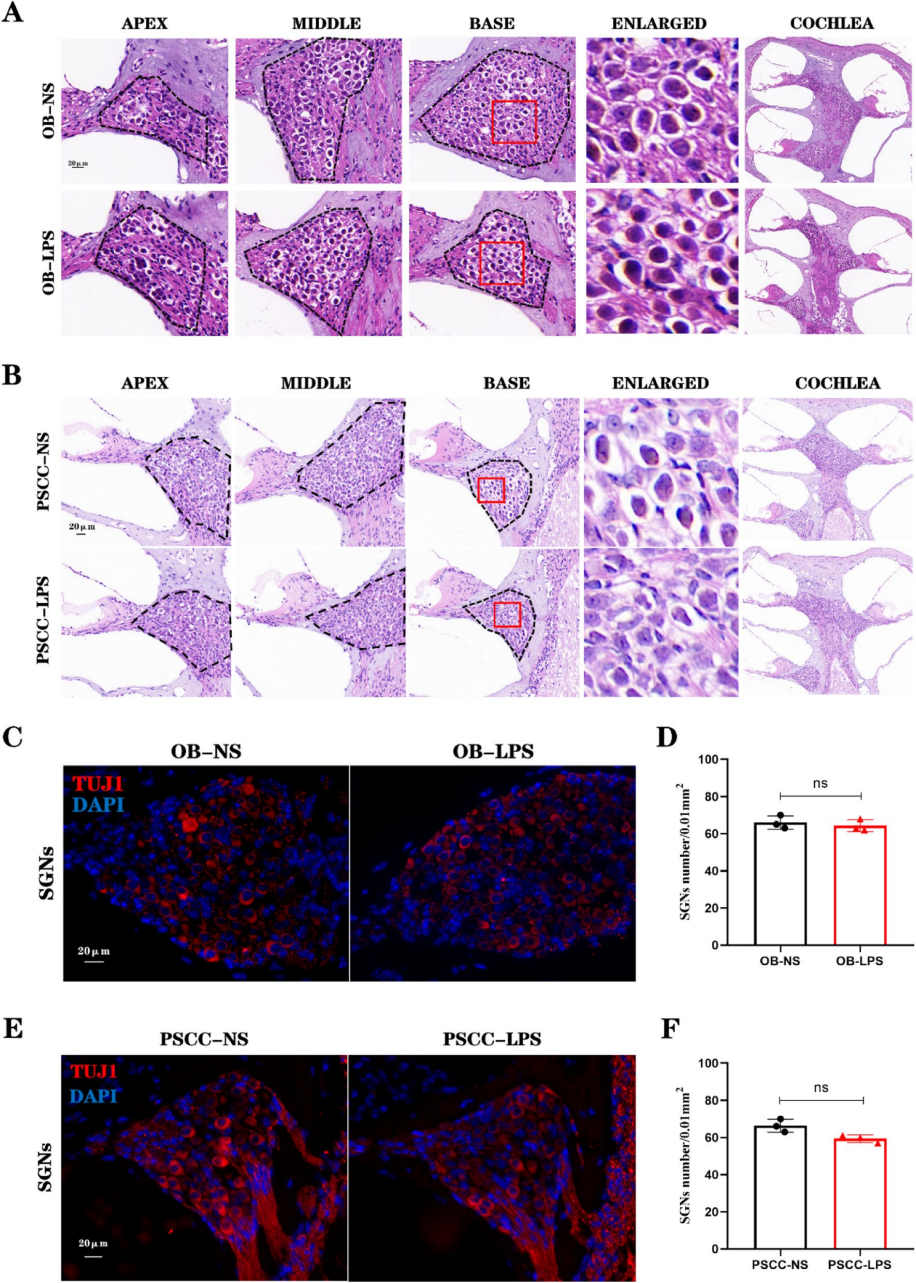
LPS Injection into the PSCC Induces damage to the SGNs. **A** No significant atrophy was observed in the SGNs of the cochlea after LPS injection into the OB. The black dashed line outlines the spiral ganglion. (*n* = 3, biological replicates, each with 3 technical replicates). **B** Atrophy was observed in the SGNs of the cochlea after LPS injection into the PSCC. (*n* = 3, biological replicates, each with 3 technical replicates). **C** TUJ1 and DAPI staining of mouse cochlear SGNs after LPS injection into the OB. **D** No significant changes in SGNs were observed after LPS injection into the OB. **E** TUJ1 and DAPI staining of mouse cochlear SGNs after LPS injection into the PSCC. **F** A reduction in SGNs was observed after LPS injection into the PSCC, however, no statistically significant difference was detected. Scale bar: 20 μm. All data are presented as mean ± SD. ns: no statistical significance

SGNs play a pivotal role in auditory signal conduction. To assess morphological changes in SGNs, HE staining of paraffin sections was performed, and immunofluorescence staining using TUJ1 antibody was conducted. HE staining revealed no significant morphological alterations in SGNs between the NS group and the LPS group following OB injection (Fig. 5A). In contrast, SGNs in the PSCC injection group exhibited significant atrophy (Fig. 5B). Immunofluorescence staining further demonstrated no substantial loss of SGNs in the OB injection group, with no significant difference in cell quantification within the spiral ganglion region (ns) (Fig. 5C-D). In the PSCC injection group, although a reduction in SGNs was observed in the basal turn after LPS injection, the difference between the two groups was not statistically significant (Fig. 5E-F).

Tissue observations of hair cells, stria vascularis, and spiral ganglia collectively indicated that LPS injection into the PSCC induced significant damage to sensorineural tissues. In contrast, LPS injection into the OB did not result in notable sensorineural injury. These findings suggest that LPS injection into the PSCC effectively simulates an inflammation-induced cochlear injury model.

### TLR4-KO mitigates LPS-induced hearing loss

**Fig. 6:**
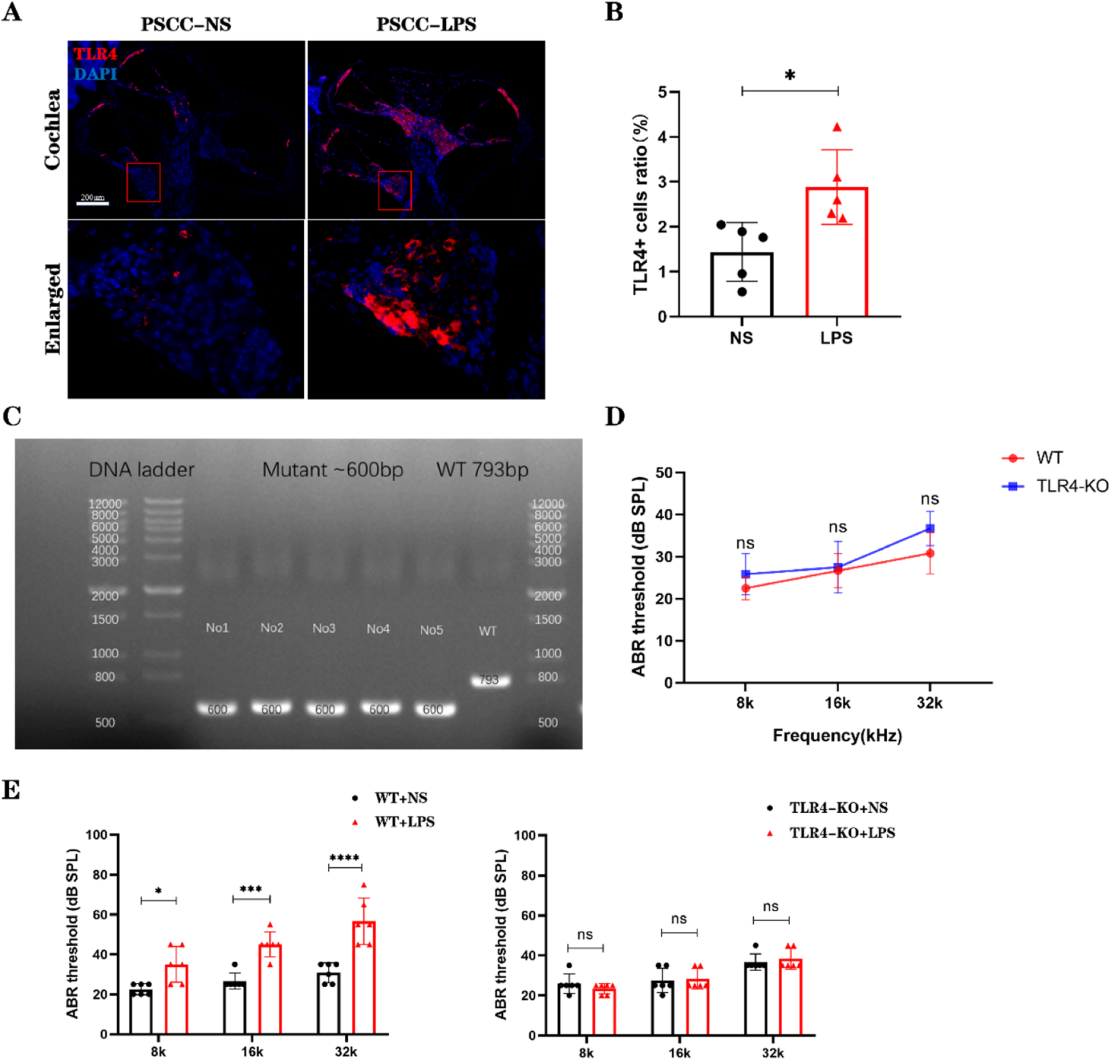
TLR4-KO mice mitigate LPS-Induced hearing Loss. **A** Expression of TLR4 in the cochlea after saline and LPS injections into the PSCC. Scale bar: 20 μm. **B** Significantly increased numbers of TLR4-positive cells were observed in the cochlea after LPS injection. (*n* = 5, biological replicates) **C** Nucleic acid gel electrophoresis results for genotyping of five randomly selected offspring mice and one WT mouse. (*n* = 5, biological replicates) **D** No significant differences in hearing thresholds were observed between TLR4-KO and WT mice under baseline conditions. (*n* = 3, biological replicates) **E** WT mice exhibited significant threshold shifts in hearing after LPS injection, whereas TLR4-KO mice showed no significant changes in hearing thresholds following LPS injection. (*n* = 6, biological replicates). All data are presented as mean ± SD. *p < 0.05, ***p < 0.001, ****p < 0.0001; ns: no statistical significance

The TLR4 receptor plays a pivotal role in the LPS signal transduction pathway. To investigate its involvement in cochlear inflammatory injury, we analyzed the expression of the TLR4 receptor in the cochlea following LPS stimulation. Immunofluorescence staining using TLR4-specific antibodies revealed that TLR4 receptors were predominantly expressed in the stria vascularis, basement membrane, and spiral ganglia of the cochlea (Fig. 6A). Moreover, significantly increased numbers of TLR4-positive cells were observed in the cochlea after LPS injection (Fig. 6B). To further evaluate the role of the TLR4 receptor in LPS-induced cochlear damage, we utilized TLR4-KO mice. Genotyping was performed on 5 offspring of genetically modified mice and 1 WT mouse. Nucleic acid gel electrophoresis confirmed that all 5 genetically modified mice were TLR4-KO (Fig. 6C). To assess whether TLR4 gene knockout affects baseline hearing, we measured ABR thresholds of mature TLR4-KO mice (aged 6–8 weeks). The ABR thresholds at 8 kHz, 16 kHz, and 32 kHz in TLR4-KO mice showed no significant difference compared to WT mice (ns) (Fig. 6D). Subsequently, we evaluated the hearing of TLR4-KO mice after LPS injection into the PSCC. The results showed no significant change (ns) in the average ABR thresholds at 8 kHz, 16 kHz, and 32 kHz frequencies in TLR4-KO mice compared to the post-surgery. In contrast, WT mice exhibited significant increases in ABR thresholds at the same frequencies following LPS injection (**** *p* < 0.0001) compared to the NS injection group (Fig. 6E).

### TLR4-KO mitigates cochlear sensory neural tissue damage

**Fig. 7:**
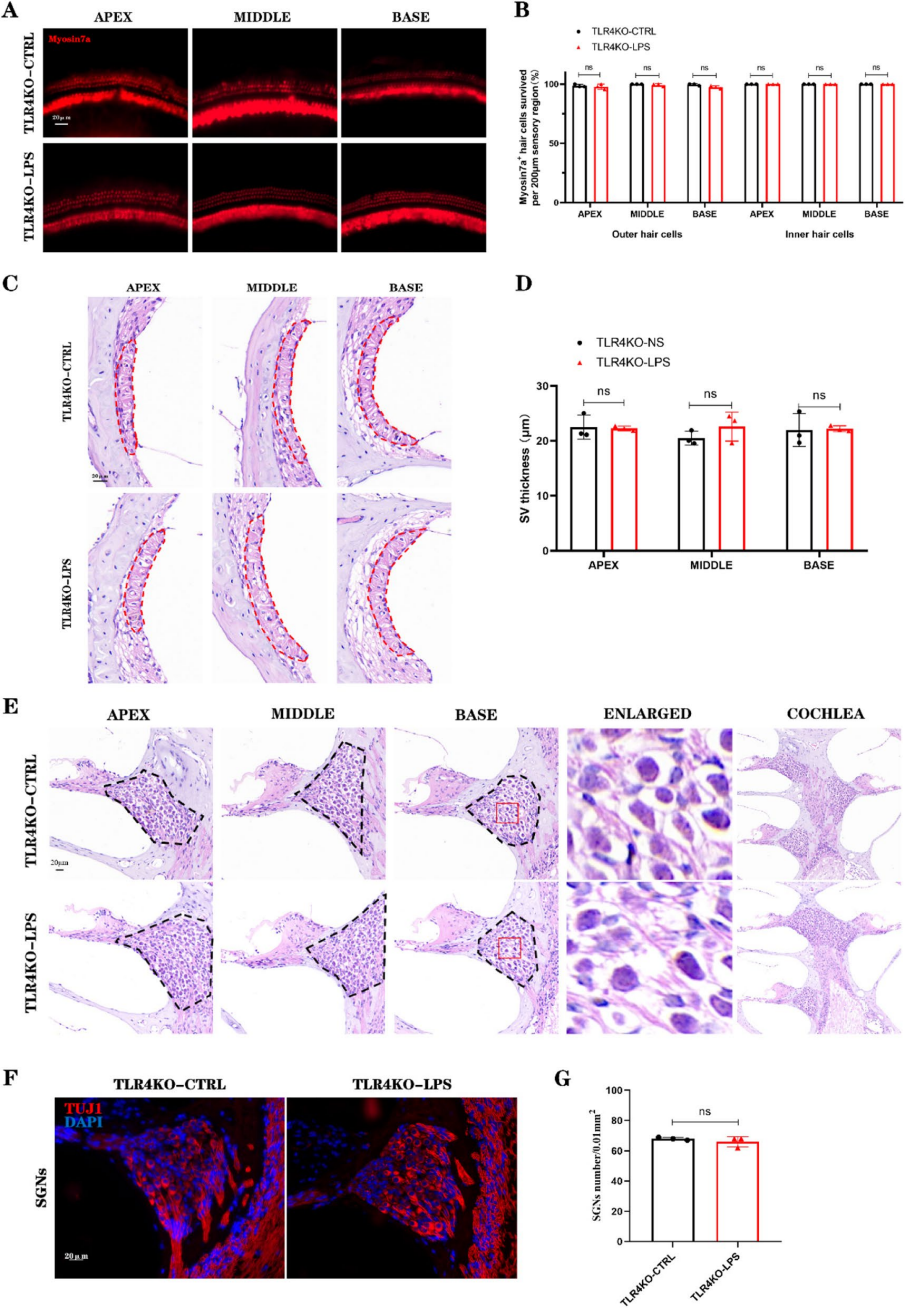
TLR4-KO alleviates cochlear tissue damage. **A** Hair cell-specific staining in the PSCC injection group of TLR4-KO mice. **B** No significant loss of hair cells was observed in the apical, middle, or basal turns of the basilar membrane in TLR4-KO mice. **C** Morphology of the stria vascularis in the cochlear lateral wall of the PSCC injection group in TLR4-KO mice, with the red dashed line outlining the stria vascularis. **D** No significant atrophy of the stria vascularis was observed in the cochleae of TLR4-KO mice. **E** No significant atrophy of SGNs was observed in the PSCC injection group of TLR4-KO mice, with the black dashed line outlining the spiral ganglion. **F** No significant loss of SGNs was observed in the PSCC injection group of TLR4-KO mice. Scale bar: 20 μm. (*n* = 3, biological replicates, each with 3 technical replicates, all data are presented as mean ± SD, ns: no statistical significance)

Seven days after LPS injection into the PSCC of TLR4-KO mice, no significant changes in hearing were observed. To evaluate hair cell loss in the cochlea, immunofluorescence staining was performed using the hair cell-specific marker Myosin7a (Fig. 7A). In the NS injection group, hair cells were neatly arranged and intact, with no evidence of significant hair cell loss. In the LPS injection group, hair cell loss in the apical turn was less than 2.2%, with no significant difference compared to the NS injection group (ns). Similarly, hair cell loss in the middle turn was less than 1%, and in the basal turn, hair cell loss was approximately 2.5%, but neither difference reached statistical significance (ns) (Fig. 7B). HE staining of paraffin sections from TLR4-KO mice revealed no significant atrophy of the stria vascularis in the apical, middle, or basal turns following LPS injection (Fig. 7C). Additionally, there were no significant differences in stria vascularis thickness between two groups (ns) (Fig. 7D). To determine whether TLR4-KO mitigates damage to SGNs, we assessed morphological changes in SGNs using HE staining of paraffin sections and immunofluorescence staining with TUJ1 antibody to label SGNs. HE staining revealed no significant atrophy of SGNs in the LPS injection group (Fig. 7E). Similarly, immunofluorescence staining showed no significant difference in SGN counts between two groups (Fig. 7F-G). These findings demonstrate that TLR4 gene knockout effectively prevents LPS-induced damage to SGNs and hair cells, supporting its protective role against cochlear inflammatory injury.

### TLR4-KO decreases the number of IBA1-positive macrophages in the cochlea

**Fig. 8:**
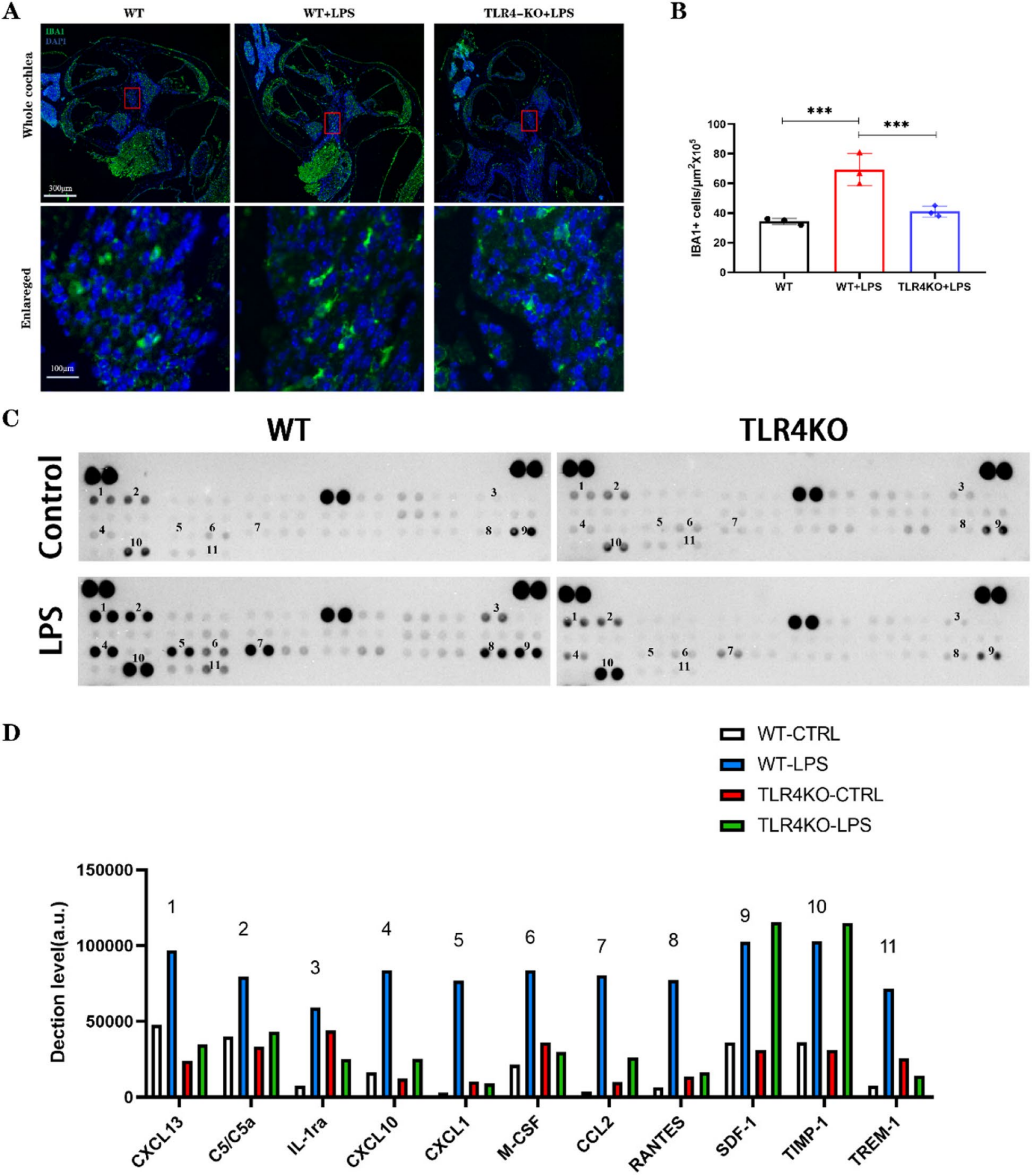
TLR4-KO mice inhibit the increase of IBA1-positive macrophages and the expression of macrophage-associated Factors. **A** Immunofluorescence staining for IBA1 in paraffin sections of the cochlea. Morphological changes in cochlear IBA1^+^ macrophages were observed after LPS injection in WT mice, which were inhibited in the TLR4-KO group. Scale bar: 300 μm. **B** An increased number of IBA1^+^ macrophages in the cochlea was observed after LPS injection in WT mice, whereas the TLR4-KO group exhibited a reduced number of cochlear IBA1^+^ macrophages. (*n* = 3, biological replicates, all data are presented as mean ± SD. ***p < 0.001) **C** Cytokine expression levels in cochlear tissue of WT and TLR4-KO mice 48 h after LPS injection into the PSCC, compared to cochlear tissue without LPS injection. **D** Quantitative analysis of differences in macrophage-related cytokines between groups, where grayscale values represent cytokine expression levels

Macrophages play a critical role in regulating inflammation as key immune cells, with the IBA1 antibody commonly utilized as a macrophage marker. To investigate the impact of TLR4 gene knockout on cochlear macrophages, we conducted slice staining on the cochleae of WT and TLR4-KO mice to examine the distribution of IBA1^+^ cells (Fig. 8A). In WT mice, IBA1^+^ cells were predominantly distributed in the spiral ligament, stria vascularis, and spiral ganglion, with sparse localization in the neural fibers of the modiolus under control conditions. Following LPS injection, a significant increase in IBA1^+^ cell numbers was observed across all cochlear regions in WT mice (**** *p* < 0.0001), accompanied by pronounced morphological changes, such as the development of dendritic protrusions. In contrast, TLR4-KO mice exhibited a marked reduction in IBA1^+^ cell numbers compared to WT mice (**** *p* < 0.0001) (Fig. 8B). These findings indicate that LPS-induced activation of IBA1^+^ macrophages in the cochlea are mediated through the TLR4 receptor.

### TLR4-KO mitigates LPS-induced cochlear inflammation

To investigate LPS-induced cochlear inflammation and assess whether TLR4-KO can mitigate this response, we analyzed cytokine secretion during inflammatory activation in cochlear tissues and examined changes in cytokine expression following TLR4-KO. Cytokine expression in cochlear tissues from WT and TLR4-KO mice was compared 48 h after LPS injection into the PSCC and under control conditions without LPS injection (Fig. 8C). Significant differences in the expression levels of multiple cytokines were observed. These cytokines include C-X-C motif chemokine ligand 13 (CXCL13), C5/C5a, IL-1ra, C-X-C motif chemokine ligand 10 (CXCL10), C-X-C motif chemokine ligand 1 (CXCL1), Macrophage Colony-Stimulating Factor (M-CSF), C–C motif chemokine ligand 2 (CCL2), RANTES, Stromal cell-derived factor 1 (SDF-1), TIMP-1, and Triggering Receptor Expressed on Myeloid Cells 1 (TREM-1). A quantitative analysis of the gray values of these differentially expressed cytokines between groups was performed (Fig. 8D). Among them, cytokines such as CXCL13, C5/C5a, CXCL10, CXCL1, M-CSF, CCL2, and RANTES, which are critical for recruiting and activating macrophages, exhibited significantly decreased expression levels in TLR4-KO mice. In contrast, SDF-1 and TIMP-1, which showed increased expression following LPS stimulation, did not exhibit a significant decrease in expression in TLR4-KO mice.

## Discussion

Developing a cochlear infection model poses significant challenges, particularly in effectively delivering infectious agents into the cochlea (Liu and Yang 2022; 2018). Currently, direct injection into the middle ear tympanic cavity and cochlea remains the primary method for local drug administration to the cochlea. In animal models where LPS is injected into the OB, ABR audiometry indicates hearing loss in mice, although pathological analyses reveal no significant cochlear damage. This finding suggests that LPS injection into the middle ear tympanic cavity may alter middle ear tissues, thereby affecting auditory conduction. The observed parallel increase in ABR thresholds at 8, 16, and 32 kHz in mice receiving LPS injections into the tympanic cavity supports the hypothesis of conductive hearing loss, which aligns with previous studies (Chai et al. 2021; Zhang et al. 2015; Choi et al. 2012). Additionally, we noted changes in the stria vascularis of the basal turn of the cochlea in mice that received LPS injections into the OB, suggesting that LPS injection into the middle ear can also induce sensorineural hearing loss, partly due to damage to the stria vascularis. However, the OB injection model, which involves injecting LPS into the middle ear tympanic cavity, has inherent limitations. First, drugs introduced into the tympanic cavity can drain through the Eustachian tube, reducing the contact time with cochlear structures such as the round window membrane and potentially hindering full penetration into the cochlea (Salt and Plontke 2018). Second, the round window membrane, composed of outer and inner epithelial cells, fibroblasts, collagen, and elastic fibers, functions as a semi-permeable barrier with limited permeability. Consequently, larger molecules or charged proteins may not effectively penetrate the cochlea (Kelso et al. 2015; Goycoolea and Lundman 1997). These factors render the OB injection model unstable due to the uncontrollable and inconsistent entry of LPS into the cochlea.

With advancements in osmotic pumps and pharmacokinetics research, semicircular canal labyrinth injection has emerged as a novel method for cochlear drug administration, offering enhanced drug delivery efficiency while minimizing cochlear tissue damage associated with intubation (Zhao et al. 2022). In our hearing tests following PSCC LPS injection, we observed that the animals'hearing stabilized after 7 days, with some improvement compared to 3 days post-surgery. Previous studies have demonstrated that the cochlea possesses homeostatic regulation capabilities that help mitigate acute damage (Crumling and Saunders 2007; Spassova et al. 2004). Accordingly, in subsequent experiments, we analyzed mice 7 days after surgery. Statistical analysis of ABR test results revealed that hearing loss was most pronounced at mid-to-high frequencies, specifically at 16 kHz and 32 kHz, consistent with basilar membrane hair cell counts. During the development of the PSCC injection model, we found that strictly limiting the surgical duration to approximately 20 min effectively prevented cochlear damage and hearing changes associated with the procedure itself. This was confirmed by the absence of significant hearing changes in the NS injection group. HE staining of cochlear sections revealed significant atrophy of the stria vascularis throughout the cochlea following LPS PSCC injection. This damage facilitated LPS entry into the cochlea, disrupting the cochlear microenvironment and its homeostasis. Staining of SGNs further revealed atrophy in this region and a marked reduction in spiral nerves connected to hair cells, indicating impaired neural conduction between hair cells and spiral nerves. Compared to LPS injection into the middle ear, PSCC injection caused more severe cochlear damage, making it a more suitable model for studying infectious cochlear injury.

TLR4 is a key molecule in the LPS signaling pathway, responsible for recognizing and binding to LPS and initiating the activation of downstream signaling molecules. This process ultimately activates transcription factors such as NF-κB, leading to the release of inflammatory mediators, including cytokines, chemokines, and reactive oxygen species (ROS) (Park and Lee 2013). These inflammatory substances accumulate in the cochlea, causing tissue damage that can result in sensorineural hearing loss (Liu et al. 2024). Our findings demonstrate that knocking out the TLR4 receptor significantly alleviates LPS-induced damage to the sensory neural tissue in the cochlea.

Macrophages are the primary immune cells in the cochlea. Under normal physiological conditions, these macrophages are predominantly located in the spiral ligament and stria vascularis within the cochlea’s lateral wall (Sato et al. 2008). Previous studies have shown that LPS can activate macrophages surrounding the stria vascularis, disrupting the blood-labyrinth barrier and increasing the permeability of the stria vascularis (Jiang et al. 2019). Additionally, macrophages are widely distributed in cochlear neural tissues, where they exhibit dendritic protrusions that resemble nerve fibers. These macrophages are thought to play a role in the development of auditory nerves (Brown et al. 2017). Consistent with these findings, our results indicate that, under control conditions, cochlear macrophages are primarily located in the stria vascularis and spiral ligament. After LPS stimulation, however, there is a significant increase in macrophage distribution within the cochlear nerve fibers of the modiolus, accompanied by more prominent dendritic protrusions. The specific role of macrophages in spiral nerves, however, remains to be further investigated and confirmed. In the basilar membrane region, macrophages are positioned on the scala tympani side of the basilar membrane and are not directly connected to hair cells. Interestingly, Hu et al. reported that the number and morphology of macrophages vary across the apical, middle, and basal turns of the basilar membrane. Macrophages at the apical turn exhibit a dendritic shape with small cell bodies and long branches, whereas macrophages near the middle turn resemble amoebas with larger cell bodies and shorter, thicker branches. Macrophages at the basal turn are round, with few or no dendritic protrusions (Hu et al. 2018). Our findings on hair cell damage revealed that hair cells in the middle and basal turns are more susceptible to loss. Whether this susceptibility is related to changes in the morphology and function of macrophages in the basilar membrane requires further investigation. During LPS-induced cochlear injury, macrophages undergo significant quantitative and morphological changes. However, our current experiments demonstrate only a correlation between LPS-induced changes in cochlear macrophages via TLR4 receptors and sensorineural hearing loss. The specific functions of the increased and activated macrophages in the cochlea remain unclear and require further research for clarification.

Cytokines are crucial regulators of the immune response and inflammatory processes, modulating the function and activity of macrophages (Ramirez et al. 2019; Sain et al. 2024). Macrophages, in turn, regulate immune responses by releasing cytokines, forming a complex network of interactions (Mitsui et al. 2023). This intricate relationship plays a pivotal role in maintaining immune system balance and preventing disease onset. Our cytokine detection results suggest that macrophage-related factors such as ICAM-1, CXCL1, CCL2, CXCL9, CXCL10, RANTES, and TIMP1 may serve as potential targets for mitigating cochlear damage. Further in-depth research is required to validate these factors as potential intervention points for future therapeutic strategies.

Our preliminary research highlights that TLR4-KO significantly reduces LPS-induced cochlear tissue damage. These findings suggest that the TLR4 receptor may serve as a potential therapeutic target for the clinical treatment of infectious cochlear injuries.

## Conclusions

The injection of LPS into the PSCC induces alterations and loss of cochlear hair cells, the stria vascularis, and SGNs, resulting in sensorineural hearing loss at medium to high frequencies. This method of LPS injection proves to be a more effective approach for establishing an animal model of cochlear inflammatory injury. Knockout of the TLR4 gene significantly attenuates LPS-induced proliferation of IBA1-positive cells in the cochlea, as well as the associated tissue damage and sensorineural hearing loss. These findings highlight TLR4 as a promising therapeutic target for the treatment of sensorineural hearing loss.

## Data Availability

No datasets were generated or analysed during the current study.
